# Warm Injections in Uterine Hæmorrhages

**Published:** 1875-04

**Authors:** 


Warm Injections in Uterine Hæmorrhages.—The success of an American physician induced Dr. Windelban, of Berlin, (*Allg. Med. Central-Zeitung*) to make use of hot injections in a case of dangerous hemorrhage in an abortion, and in which case the midwife in attendance had already resorted to ergot and cold injections without at all checking the loss of blood. The effect was immediate, and was all that could be desired. Within a few seconds after the entrance of the hot water, such energetic contractions of the womb came on, that the fœtus, which had just presented at the os, but which was almost beyond reach, was, together with the secundines, expelled within a quarter of an hour. Encouraged by the energetic excitation of the uterine muscular tissue, the injections were repeated during the following days, whenever any signs of recurrence of the hemorrhage manifested themselves; they were continued until the normal inversion of the uterus had been accomplished, but the temperature of the water was gradually lowered until lukewarm water was used. The author has employed the warm injections with the very best results, not only in 11 abortions, but in all kinds of uterine hemorrhages, in 2 cases of profuse hemorrhage accompanying placenta prævia, in that caused by fibroids and other tumors in the cavity and in the walls of the uterus, in carcinoma of that organ, in hemorrhage from the empty and relaxed uterus after parturition, in profuse menstruation, in short, in all cases of uterine hemorrhage which came under his care during the past year. The injections were always practised by means of the simple uterine douche, after the manner of irrigation, the water being of a temperature of 34°-38°C. (92°-100° Fahr.); no ill effects, but on the contrary, the greatest advantage was always observed after their use.

